# Parenting style and child mental health at preschool age: evidence from rural China

**DOI:** 10.1186/s12888-024-05707-1

**Published:** 2024-04-24

**Authors:** Lei Wang, Jing Tian, Scott Rozelle

**Affiliations:** 1 International Business School, Shaanxi Normal University, Xi’an, Shaanxi China; 2 School of Economics and Management, Shaanxi Xueqian Normal University, Xi’an, Shaanxi China; 3 Stanford Center on China’s Economy and Institutions, Stanford University, Stanford, USA

**Keywords:** Parenting styles, Mental health problems, Children at preschool age, Rural China

## Abstract

**Background:**

Mental health problems among children at preschool age are a common issue across the world. As shown in literature, a caregiver’s parenting style can play a critical role in child development. This study aims to examine the associations between a caregiver’s parenting style and the mental health problems (or not) of their child when he/she is at preschool age in rural China.

**Methods:**

Participants were children, aged 49 to 65 months, and their primary caregivers. The primary caregivers of the sample children completed the Parenting Styles and Dimensions Questionnaire, Short Version, the Strengths and Difficulties Questionnaire, and a questionnaire that elicited their socio-demographic characteristics. The level of cognitive development of each sample child was assessed using the Wechsler Preschool and Primary Scale of Intelligence, Fourth Edition. Pearson correlation analysis, linear regression analysis, and multivariable regression analysis were used to analyze the data.

**Results:**

The prevalence of mental health problems among sample children at preschool age was high (31.6%). If a caregiver practices an authoritative parenting style, it was found to be negatively associated with the mental health problems of their child. In contrast, a caregiver’s authoritarian parenting style was positively associated with the mental health problems of their child. Compared to those in a subgroup of primary caregivers that used a combination of low authoritative and low authoritarian parenting style, primary caregivers that used a combination of high authoritarian and low authoritative or a combination of high authoritative and high authoritarian were found to have positive association with child health problems. A number of demographic characteristics were found to be associated with the adoption of different parenting styles.

**Conclusion:**

Different parenting styles (including authoritative, authoritarian, and combination of authoritative and authoritarian) of the sample caregivers had different associations with the mental health problems of the sample children. Parenting programs that aim to improve the parenting styles (favoring authoritative parenting styles) should be promoted in an effort to improve the status of child mental health in rural China.

**Supplementary Information:**

The online version contains supplementary material available at 10.1186/s12888-024-05707-1.

## Introduction

Mental health problems of young children have become a common public health issue worldwide in recent years [1, 2]. In this paper, “mental health problems in early childhood” are those problems that involve normative issues of emotions, behavior and social interactions, which can be generally categorized into three broad categories: internalizing problems (e.g., depression, withdrawal, anxiety, somatization); externalizing problems (e.g., aggression, oppositional defiance, attention deficit, hyperactivity); and social problems (e.g., difficulties in socializing with others) [3–5]. A growing literature has indicated that the incidence and prevalence of mental health problems among preschool children is relatively high across the world [6–8]. Data from developed countries estimate that approximately 4–19% of preschool-aged children are experiencing mental health problems such as attention disorders, hyperactivity and/or emotional or behavioral problems [9–11].

In developing countries, the prevalence of mental health problems among preschool-aged children is even higher [12–14]. A study conducted in Brazil showed that 24% preschool-aged children were found to have mental health problems [12]. In South Africa, the prevalence of mental health problems among young children is much higher: 36.7% children aged 4–6 were identified to be abnormal in the state of mental health [14]. In the recent literature in China, researchers have started to examine the mental health problems among young children. Evidence has shown that child mental health problems at preschool age are prevalent in China, especially in rural areas [15–18]. Studies conducted in urban China have revealed that, approximately, 5–20% of preschool children are suffering from some type of mental health problems [19–21]. The rate of mental health problems among preschool children in rural China is even higher, up to 39% [17, 18]. In a study by Li et al. (2021), around 70% of rural preschoolers were identified with at least one kind of mental health problems. Specifically, the rates of emotional problems, conduct problems, hyperactive/inattention problems, peer relationship problems, and prosocial behavioral problems were 39%, 27%, 23%, 12% and 26%, respectively [17].

In addition to the issues that the preschool children and their families experience during the preschool years, the literature also shows that mental health problems that occur when children are preschool age can have long-lasting negative effects on the children as they age, including problems such as poor levels of school readiness, psychological well-being and social relationships [22–24]. Untreated mental health problems at preschool age can lead to chronic mental disorders or more serious long-term behavioral problems, such as learning difficulties, school dropout, substance abuse, domestic violence, and even suicide [4, 7, 25]. Because of these longer-run possible consequences, it is thus critical to identify the mental health problems and conduct interventions that are able to solve mental health problems of children at preschool age [26–28].

### Parenting style and child mental health

Since the strategies and approaches that caregivers use in their parenting activities when rearing their young children (e.g., 0 to 3 years old) have significant effects on child mental health outcomes, poor parenting styles have been identified as a primary risk factor of mental health problems among children [29–31]. As a central form of socialization that shapes the development of children, the family is a socio-cultural-economic arrangement that exerts significant influence on the behavior and character of children and thus parents play a considerable role in the development of children, especially during the early years of childhood [32–35]. In this sense, parenting styles can have important and lasting impacts on child development. Any inappropriate parenting styles such as physical punishments may lead to unwanted damaging effects on the mental health of children [32, 36, 37]. 

According to Baumrind (1967), caregivers do have choices as parenting styles can be divided into three categories: authoritative, authoritarian, and permissive. Authoritative parenting is characterized by high levels of warmth, responsiveness, encouragement of the autonomy of children, and democratic disciplinary strategies. In contrast, authoritarian caregivers often parent their children through physical coercion, verbal hostility, and punishment without explanation. Permissive parenting involves a combination of high warmth and acceptance and low expectations of the child as he/she is maturing [38]. Based on these categories, a scale, the Parenting Styles and Dimensions Questionnaire (PSDQ), was created to assess the style of parenting in the context of different cultural settings [39].

Research has found that different categories of parenting style measured by the PSDQ have shown different associations with the emergence of mental health problems of their children [31, 40, 41]. Specifically, an authoritative parenting style has been shown to have negative associations with the mental health problems of preschool children. In contrast, authoritarian and permissive parenting styles have been observed to be positively associated with mental health problem of children at preschool age. For example, a study of families in the United States demonstrated that, when compared to authoritarian and permissive parenting styles, adopting an authoritative parenting style was most predictive of fewer behavior problems of preschool children [31]. Another national cohort study conducted in the United Kingdom found that the attitudes of caregivers that adopted authoritarian style to discipline their children were associated with the occurrence of mental health problems of children at the age of 5 [41]. Research conducted by Hanafi & Thabet (2017) illustrated that, in the Gaza Strip, authoritative parenting style was negatively associated with mental health problems of preschool children. In contrast, children with authoritarian or permissive parenting style caregivers were more likely to have mental health problems [40].

Research also has investigated the reasons why different patterns of parenting styles have been shown to have different degrees of associations with the mental health problems of young children [31, 40–47]. Previous studies have shown that warm and responsive parenting behavior are consistently associated with developmental outcomes of early childhood, including mental health; in contrast, the children of parents that displayed low levels of warmth showed elevated levels of oppositional behavior [42, 48, 49]. Since authoritative parents care more about the feelings of their children, and give more encouragement to their children, this behavior not only provides psychological support but also autonomy in various activities; in short, children raised by authoritative parents can express their ideas freely and can create their own self-confidence and responsibility with less problematic behavior [36, 46, 47, 49]. In contrast, authoritarian parents are not only often unresponsive to the needs of their children but also frequently are demanding. Parenting strategies, such as punishments, the use of forces and harshness are mostly used by authoritarian parents, and these actions often result in disobedient behavior, aggression, and restlessness of children, which can ultimately be detrimental to the mental health of children [43, 45, 48, 49]. According to Baumrind (1967; 2013), permissive parents do not guide their children to regulate their behavior; instead, permissive parents tend to allow their children to make their decisions alone. Therefore, the children of parents that us a permissive style of parenting often become dependent and lack social responsibility, exhibiting either internalizing or externalizing behavior problems [32, 38, 40, 49].

Previous studies have also revealed that certain demographic characteristics of children and households are associated with the adoption of different parenting styles [30, 50–52, 44, 49, 53–55]. When examining child characteristics, a strand of literature has explored the differences of parenting styles that are used with children of different gender [30, 51, 55–57]. The results of these studies show that parents tend to use authoritarian parenting style for boys more frequently and tend to use authoritative parenting style for girls. Other studies have also examined the associations between the number of children in a household and the parenting style that was adopted by the caregiver [44, 54]. Comparing to families with only one child, parents are more likely to use authoritarian parenting styles than when they have more than one child. When examining household characteristics, previous studies have frequently focused on the association between parenting style and the socioeconomic status (SES) of the parents [50, 52, 53]. According to these studies, parents with higher levels of SES (e.g., higher levels of education and higher incomes) are more inclined to use authoritative parenting style. In contrast, parents with lower levels of SES are more likely to adopt authoritarian parenting style.

### Parenting style and mental health of preschool children in China

Literature in China has also begun to investigate the sources of mental health problems among preschool children in China [15, 58, 59]. Similar to the findings of the literature on this topic outside of China, studies in China show that the type of parenting style used by the caregiver is one of the primary sources of the mental health problems of their children. For example, a study conducted in urban preschools of Guangzhou found that the adoption of authoritarian parenting style resulted in an increase in child mental health problems [15]. In contrast, the findings of another study conducted in a city located in the central region of China suggest that authoritative parenting style actually improved the mental health outcomes of preschool children [58].

### The current study

Literature on parenting styles of parents in China have showed that more and more urban parents are adopting authoritative parenting style rather than relying on authoritarian parenting style as they are rearing their children—or at least are relying on more blended forms of authoritative/authoritarian parenting styles [60–65]. Importantly, however, to our knowledge there has never been a study on the adoption of parenting styles of caregivers that are raising preschool-aged children in rural China.

In addition, even though there have been studies on the association between parenting styles and mental health problems of preschool children in China, most of studies have been conducted with urban children and their parents [15, 58, 59]. Considering the great difference in child-rearing practices and the nature of child development between urban and rural areas, it is of great significance to understand the associations between parenting styles and the levels of mental health of children at preschool age in rural China. Examining the context of rural China is important because in studies of preschool children in rural China it has been found that rural China has much higher levels of mental health problems compared to their urban peers. As discussed previously, since mental health problems during early childhood can have long-lasting negative effects on children in many aspects, it is important to address the issue of high prevalence of mental health problems among preschool children in rural China. Since improving caregiver-child interactions can mitigate the mental health problems of children, understanding the associations between parenting styles and child mental health might encourage caregivers in rural China to adopt parenting strategies that improve the outcomes of child mental health. To the best of our knowledge, however, no related work with children at preschool age has been conducted in rural areas of China. The current study aims to fill this gap to the literature.

Given the absence of literature on parenting style and mental health of preschoolers in rural China, the current study uses data drawn from a longitudinal study to examine the association between different parenting styles and the levels of mental health of children at preschool age in rural China. To achieve this goal, the current study has four specific objectives. First, the study examines the state of parenting styles and mental health outcomes of children at preschool age in rural China. Second, the study investigates the associations between different parenting styles and the levels of mental health of rural children at preschool age. Third, the study explores the associations between different subgroups of combined parenting styles and mental health of preschool aged children in rural China. Finally, the study identifies some of the correlates—both child characteristics and household characteristics—of the adoption of different parenting styles.

The remainder of this paper is organized as follows. The next section describes the methods that we used. The third section presents the results of our analysis. The final two sections discuss the findings and conclude.

## Methods

### Participants

The data for the current study are drawn from a longitudinal study of children and households conducted in 11 nationally designated poverty counties in northwestern China. The selection of the sample for this longitudinal study was conducted in 2013 and followed a multistage clustering sampling design. First, all townships (n = 174) in the 11 study counties were included in the study. Next, we randomly selected two villages from each of the sample townships. In total, 351 villages were included. Finally, after excluding children with known diseases or disabilities, we selected all of the remaining children that were within the target age range (6–12 months) for inclusion in the longitudinal study. Overall, 1,802 children were enrolled into the study. After the sample selection, we conducted the baseline survey. In the following years, we followed up with the sample children and households several times after the initial baseline. In 2017, when the sample children were 49–65 months age, we conducted the most recent follow-up survey and tracked 1,459 children and their caregivers from 351 villages successfully. All the participants included in the current study completed a follow-up survey in 2017 (when the children were preschool age). Table 1 shows the demographic characteristics of children and households that were part of the 2017 survey.

**Table 1:** Descriptive statistics

	Frequency (n)	Percentage/M ± SD
***Child Characteristics***		
Child age (months)	1459	56.87 ± 3.43
Sex		
Male	751	51.47%
Female	708	48.53%
Has siblings		
Yes	665	45.58%
No	794	54.42%
Attends preschool		
Yes	1375	94.24%
No	84	5.76%
Left-behind child		
Yes	359	24.61%
No	1100	75.39%
Delayed in cognitive development		
Yes	650	44.55%
No	809	55.45%
***Household Characteristics***		
Mother is primary caregiver		
Yes	903	61.89%
No	556	38.11%
Primary caregiver age (years)	1459	34.31 ± 10.00
Primary caregiver education		
< 9 years of schooling	493	33.79%
≧ 9 years of schooling	966	66.262%
Family asset index	1459	-0.00 ± 1.21

Note. Children with scaled index values more than one SD below the mean are considered developmentally delayed

### Measures

#### Parenting styles and dimensions Questionnaire-Short Version (PSDQ-Short Version)

We used the Parenting Styles and Dimensions Questionnaire-Short Version (PSDQ-Short Version) to measure the parenting styles of the primary caregivers of sample children [39]. The PSDQ-Short Version is a self-report questionnaire containing 36 items. The responses are given based on a 5-point Likert scale that ranges from never (1) to always (5). The PSDQ-Short Version includes three sub-scales of parenting style: authoritative, authoritarian, and permissive. Higher scores on each scale indicate that the respondent exhibits parenting behaviors that are more consistent with that particular parenting style. The translated Chinese version of the PSDQ-Short Version has been shown to have adequate internal consistency and reliability [64, 66–68]. As several studies reported parents rarely use permissive parenting in China [62, 64] and the permissive scale was not reliable among Chinese parents [60, 65, 69], our research team did not collect data on the items for a permissive parenting style. The present study, therefore, had two subscales—authoritative and authoritarian parenting styles, with a total of 27 items. Examples of the items are as follows: “I am responsive to my child’s feelings or needs.” “I take my child’s desire into account before asking my child to do something.” “I punish by taking privileges away from my child with little if any explanations.” The items were rated on a 5-points scale (1 = Never, 2 = Once in a while, 3 = About half of the time, 4 = Very often, 5 = Always). The subscale of the authoritative parenting style contains three dimensions with 15 items: Connection (Warmth and Support), Regulation (Reasoning or Induction), and Autonomy Granting (Democratic Participation). The subscale of authoritarian parenting style is comprised of three dimensions with 12 items: Physical Coercion, Verbal Hostility, and Non-Reasoning or Punitive. In the current study, the scale of PSDQ demonstrated good internal consistency. The Cronbach’s alpha was 0.77.

### Strengths and Difficulties Questionnaire (SDQ)

The Strengths and Difficulties Questionnaire (SDQ) is a 25-item carer-reported instrument to assess mental health of children aged 2 to 17 [70]. For the current study, we used the Chinese version of SDQ that has been validated for the Chinese context [71]. Examples of the questions are: “Your child is helpful if someone is hurt, upset or felling ill.” “Your child often has temper tantrums or hot tempers.” “Your child has at least one good friend.” All items are scored on a three-point Likert scale (0 = not true, 1 = somewhat true, and 2 = certainly true). There are five subscales within the SDQ regarding three domains of mental health problems: Internalizing problems (including subscales of emotional symptoms and peer relationship problems); Externalizing problems (including subscales of conduct problems and hyperactivity problems); and Prosocial behaviors. Each subscale is further divided into three categories, namely, “normal,” “borderline,” and “abnormal” [71]. The cutoffs of the three categories for each subscale can be found in Appendix Table A1. In the current study, children with scores either in the abnormal or borderline range on the total difficulties scale and individual subscales were classified having mental health problems. The scale of the SDQ showed good reliability for our sample. The Cronbach’s alpha was 0.68.

#### Wechsler Preschool and Primary Scale of Intelligence-Fourth Edition (WPPSI-IV)

We used the Wechsler Preschool and Primary Scale of Intelligence-Fourth Edition to assess the levels of cognitive development of the sample children (WPPSI-IV; [72]). The WPPSI-IV is comprised of 13 subtests that are incorporated together to form one Full-Scale Intelligence Quotient (FSIQ). The Chinese version of WPPSI-IV was adapted in 2010 and has subsequently been used in studies across China [73, 74]. The WPPSI-IV is usually administered 1-on-1 by trained testers, using a standardized set of toys and detailed scoring sheets. In the current study, the Cronbach’s alpha for this scale was 0.91, indicating a high reliability. For the analysis of the current study, children with an FSIQ score below 85 (one standard deviation below the mean of 100) are considered to be developmentally delayed. According to the literature [75–77], in early childhood, the cognitive development of a child can promote the formation of his or her noncognitive development (including mental health). Because of this possibility of “skills begetting skills,” in the analyses of the current study, we controlled for the cognitive development of sample children (by including a measure of cognitive development as a control variable in the regression analysis).

### Socio-demographic characteristics

A primary caregiver-reported questionnaire was used to collect data on child and household characteristics. The child characteristics that are in the survey include age in months, gender, whether the child had siblings, whether the child was left-behind (i.e., either parent migrated for work at the time of the survey), and whether the child attended preschool. Household characteristics included the identity of the primary caregiver (e.g., mother or grandmother), age of the primary caregiver (years), whether the primary caregiver had obtained at least a junior high school education, and a household assets index. The household assets index was constructed using polychoric principal component analysis based on the following information: whether a household had access to tap water, a toilet with running water, a water heater, a washing machine, a computer, the Internet, a refrigerator, air conditioning, a motorcycle or electronic bicycle, and/or an automobile.

### Procedure

The data collection was carried out by enumerators recruited from local universities in 2017. Prior to the field work, the enumerators underwent a formal, week-long training course, including 2.5 days of in-the-field training. During the data collection, enumerators visited each household of the sample children individually. Upon arriving at each household, the enumerators explained the goal and content of the survey and obtained consent from the primary caregivers for both their participation and that of the child of their family that was included in the current study. The enumerators then collected data on the PSDQ and the SDQ scales as well as the information on the socio-demographic characteristics by interviewing the primary caregivers. In addition, the WPPSI-IV was administered one-on-one to each child, using a standardized set of toys and a detailed scoring sheet.

### Statistical analysis

All statistical analyses were conducted using STATA 16.0 Version. All statistical tests are two-sided. A *P*-value < 0.05 is considered statistically significant. Descriptive analyses are used to describe the sample characteristics. Continuous and categorical data are reported in the form of means (SD) and percentages. The frequencies and distributions of the status of the mental health of the sample children and parenting styles of primary caregivers are analyzed. Following two previous studies [78, 79], we sorted the children in our sample into four groups with combinations of parenting styles using the medians of scores on the authoritative and authoritarian subscales. Bivariate correlation matrices using Pearson correlation analysis is performed to determine the simple correlation between child mental health, parenting styles and different socio-demographic characteristics. Linear regression analysis is used to examine the associations between parenting styles and mental health of children. Multivariable regression analyses are also used to investigate the determinants of different parenting styles.

## Results

Table 2 shows the prevalence of mental health problems of the sample children. In our sample, over 30.0% of children were found of being at risk of having mental health problems according to the total difficulties scale. In terms of the internalizing subscales, emotional problems were more prevalent among children (34.9%) than peer problems (6.8%). In contrast, in the subscales of externalizing problems, both conduct problems (40.1%) and hyperactivity problems had high levels of prevalence (24.5%). In addition, 20.8% of children had prosocial behavior problems.

**Table 2:** Child mental health outcomes (*N* = 1,459)

		Mean score	Standard deviation	Abnormal n (%)	Borderline n (%)	Normal n (%)
Total difficulties (0–40)		12.43	4.53	261 (17.89%)	200 (13.71%)	998 (68.40%)
***Subscales of total difficulties***						
*Internalizing problems (0–20)*						
Emotional symptoms (0–10)		2.89	1.86	282 (19.33%)	227 (15.56%)	950 (65.11%)
Peer problems (0–10)		2.19	1.51	45 (3.08%)	54 (3.70%)	1360 (93.21%)
*Externalizing problems (0–20)*						
Conduct problems (0–10)		2.24	1.42	251 (17.20%)	334 (22.89%)	874 (59.90%)
Hyperactivity problems (0–10)		5.12	2.05	181 (12.41%)	177 (12.13%)	1101 (75.46%)
*Prosocial behaviors (0–10)*		7.08	1.91	133 (9.12%)	171 (11.72%)	1155 (78.16%)

*p <.05 **p <.01 *** *p* <.001

The results in Table 3 indicate that the authoritative parenting style, both according to the total scores and the scores of each dimension, rated higher than those of the authoritarian parenting style among primary caregivers in our sample. The difference in the total scores of authoritative and authoritarian parenting styles was statistically significant (*p* <.001), indicating that primary caregivers were more likely to conduct strategies related to authoritative parenting style than authoritarian parenting style.

**Table 3:** Scores of parenting styles (*N* = 1,459)

	Mean	SD
Authoritative parenting style average score	3.36	0.61
Connection dimension	3.67	0.61
Regulation dimension	3.42	0.76
Autonomy granting dimension	2.98	0.78
Authoritarian parenting style average score	2.23	0.57
Physical coercion dimension	2.19	0.71
Verbal hostility dimension	2.65	0.70
Non-reasoning/punitive dimension	1.84	0.67

The analysis using the Pearson correlations indicates that different parenting styles have different associations with the nature of the mental health of the sample children (Appendix Table A2). Regression analysis also shows that an authoritative parenting style was negatively associated with child mental health problems in the measurements of total difficulties, internalizing problems, and externalizing problems. The results also illustrate that an authoritative parenting style was positively associated with the measurement of prosocial behaviors. In contrast, an authoritarian parenting style was positively associated with child mental health problems as measured according to the scales of total difficulties, internalizing and externalizing problems. An authoritarian parenting style was negatively associated with prosocial behaviors (Table 4). Similar associations were also found between the subscales of each parenting styles and child mental health problems (Appendix Table A3). Specifically, all three subscales of the authoritative parenting style (i.e. Connection, Regulation and Autonomy granting) were significantly and negatively associated with the variables that measure: Total difficulties, Internalizing problems and Externalizing problems. In addition, all three subscales were significantly and positively associated with the variable measuring the Prosocial behavior of sample children. There is only one exception: the results show that the association between the Autonomy granting and the Internalizing problems was negative but insignificant. In regards to the three subscales of the authoritarian parenting style (i.e. Physical coercion, Verbal hostility, and Non-reasoning), positive and significant associations were found with the variables measuring Total difficulties, Internalizing problems and Externalizing problems. In addition, significant and negative associations between Physical coercion and the Prosocial behavior of children was also observed. However, no significant associations were found between the other two subscales of the authoritarian parenting style and the Prosocial behavior of children.

**Table 4:** Associations between primary caregiver’s parenting style scores and child mental health outcomes (*N* = 1,459)

	Total difficulties score	Internalizing problems score	Externalizing problems score	Prosocial behaviors score
Scores of authoritative parenting style	-0.95***	-0.34*	-0.61***	0.95**
	(0.23)	(0.14)	(0.14)	(0.09)
Scores of authoritarian parenting style	2.33***	1.16***	1.17***	-0.20*
	(0.23)	(0.14)	(0.14)	(0.10)
Controls	Yes	Yes	Yes	Yes
Cluster	Yes	Yes	Yes	Yes
County fixed effects	Yes	Yes	Yes	Yes
Tester fixed effects	Yes	Yes	Yes	Yes

Note. Each cell is a separate regression. Controls include child age, child gender, whether child has siblings, whether child attends preschool, whether child is cognitively delayed, whether child is left-behind (defined as both parents migrate), whether primary caregiver is mother, caregiver’s age, whether caregiver has obtained at least 9 years schooling, and the household asset index

*p <.05 **p <.01 *** *p* <.001

When the analysis sorted children into four groups of different combinations of “high” versus “low” values of the two parenting styles (Group 1: high authoritative, low authoritarian; Group 2: high authoritarian, low authoritative; Group 3: high authoritative, high authoritarian; and Group 4: low authoritative, low authoritarian) using ratings of primary caregivers from the authoritative and authoritarian parenting style scales, the share of each of the four groups was similar (around 25% for each) (Appendix Table A4). In the association analysis, we use Group 4 (low authoritative, low authoritarian) as the reference group. Results of the association analyses of the four groups with child mental health show that when compared to children in Group 4 (low authoritative, low authoritarian), children in Group 1 (high authoritative, low authoritarian) had significantly lower scores in the externalizing problems domain and higher scores in the domain of the prosocial behaviors (Table 5). No significant associations were found between Group 1 other two measurements of child mental health problems. In contrast, children in Group 2 (high authoritarian, low authoritative) had significantly higher scores in the scales that measured total difficulties, internalizing problems, and externalizing problems. No significant associations were found in the measurement of prosocial behaviors. Finally, children in Group 3 (high authoritative, high authoritarian) were found to have significantly higher scores in the scales that measured total difficulties, internalizing problems, and prosocial behaviors. However, no significant association was found in the measurement of externalizing problems.

**Table 5:** Associations between primary caregiver’s parenting styles at different groups and child mental health outcomes (*N* = 1,459)

	Total difficulties score	Internalizing problems score	Externalizing problems score	Prosocial behaviors score
	(1)	(2)	(3)	(4)
Group 1 (high authoritative, low authoritarian)	-0.67	-0.00	-0.66**	0.93***
	(0.35)	(0.21)	(0.21)	(0.14)
Group 2 (high authoritarian, low authoritative)	2.41***	1.13***	1.28***	-0.26
	(0.33)	(0.20)	(0.22)	(0.14)
Group 3 (high authoritative, high authoritarian)	0.88*	0.64**	0.24	0.78***
	(0.35)	(0.22)	(0.21)	(0.13)
Controls	Yes	Yes	Yes	Yes
Cluster	Yes	Yes	Yes	Yes
County fixed effects	Yes	Yes	Yes	Yes
Tester fixed effects	Yes	Yes	Yes	Yes

Note. The cutoffs of the classification are the medians of the authoritative and authoritarian subscale scores. Each cell is a separate regression. Controls include child age, child gender, whether child has siblings, whether child attends preschool, whether child is cognitively delayed, whether child is left-behind (defined as both parents migrate), whether primary caregiver is mother, caregiver’s age, whether caregiver has obtained at least 9 years schooling, and the household asset index

*p <.05 **p <.01 *** *p* <.001

When investigating the correlates of the authoritative and authoritarian parenting styles respectively (Table 6), a number of demographic characteristics of the sample children and sample households were found to be significant. The demographic characteristics that had significant correlations with the authoritative parenting style include whether the child cognitively delayed, educational level of primary caregiver, and household economic status. In contrast, many more demographic characteristics, such as child age, child gender, left-behind child, whether the child cognitively delayed, whether the mother is the primary caregiver, and household economic status, were found to have significant associations with the adoption of the authoritarian parenting style.

**Table 6:** Determinants of parenting styles (*N* = 1,459)

Variable	Authoritative parenting scores	Authoritarian parenting scores
Child age (months)	-0.00	-0.01*
	(0.00)	(0.00)
Male	-0.03	0.06*
(1 = Yes)	(0.03)	(0.03)
Has siblings	-0.03	0.05
	(0.03)	(0.03)
Attends preschool	-0.07	-0.01
	(0.10)	(0.09)
Left-behind child	-0.09	0.12*
	(0.05)	(0.05)
Delayed in cognitive development	-0.09**	0.10**
	(0.03)	(0.04)
Mother is primary caregiver	0.07	-0.13**
	(0.04)	(0.04)
Primary caregiver age (years)	0.00	0.00
	(0.00)	(0.00)
Primary caregiver’s education	0.12***	-0.07
	(0.04)	(0.04)
Household asset index	0.07***	-0.04**
	(0.01)	(0.01)

*p <.05 **p <.01 *** *p* <.001

## Discussion

Considering the importance of identifying mental health problems of young children at preschool age (or issues involving the development of their social emotional abilities), this study first investigated the prevalence of mental health problems among preschool-aged children in rural China. The results of the current study show that the prevalence of child mental health problems is high in rural China, no matter whether it was measured by the total difficulties score of the SDQ scale, or by scores on the three domains of the SDQ scale (i.e. Internalizing problems, Externalizing problems, and Prosocial behavior problems). When looking at the literature, the prevalence in rural China appears to be higher than what has been found among preschoolers in developed countries [9–11]. When comparing the results of the sample children in this study to peers in other developing countries (including comparisons to children in urban China), the sample children of the current study still show relatively higher prevalence of mental health problems [12, 14, 15]. Importantly, these results are consistent with previous studies conducted in other areas of rural China [17, 18]. One possible reason for the high prevalence of child mental health problems in rural China might be that there are many rural children are left-behind due to parental migration. Nearly one quarter (25%) of the children in the sample were left-behind children. According to previous studies, the absence of parents may have a negative impact on the mental health of the child [17, 18]. Another potential reason lies in the fact that public health services are poor in rural China which might delay the detection and timely treatment of children with mental health problems. In this sense, actions that improve both the interactions between migrant parents and left-behind children and the public health system in rural China are encouraged. Otherwise, the prevalence of child mental health in rural China likely would persistently be high or become even higher.

Previous studies have provided evidence that suggests that parenting style is a potential risk factor of child mental health problems [29–31]. The current study reveals that although the primary caregivers of the children in the sample are more likely to be using authoritative parenting style (rather than authoritarian parenting style), when compared to urban studies carried out in China, the results of this study show that the use of strategies regarding the authoritative parenting style in our sample is significantly lower than in urban China [62, 69]. In contrast, studies also show that the adoption of authoritarian parenting styles in rural China (as shown in this study) is higher than in urban China [60–65]. These findings indicate that rural caregivers appear to be increasingly influenced by their urban peers in the parenting styles due to the economic transition that China is undergoing, including trends in urbanization and rural-to-urban migration. However, continued disparities in many dimensions between rural and urban caregivers in China (e.g. economic status, educational levels, and values) are still barriers that are slowing down improvements in the adoption of parenting styles in rural families [17, 18, 69]. There is, thus, an urgent need to help rural families in China better understand the advantages of adopting an authoritative parenting style and the disadvantages of using an authoritarian parenting style. If this can be taught to rural caregivers, it is hoped that this will encourage those rural caregivers to interact with their young children more with authoritative parenting styles and less with authoritarian parenting styles.

Consistent with the literature, our study shows that the authoritative parenting style is negatively associated with the occurrence of mental health problems of children in terms of all dimensions of the SDQ scale, including Total difficulties; Internalizing problems; Externalizing problems; and Prosocial behavior problems [31, 40, 58]. As described by Baumrind (1966), children raised by authoritative caregivers grow up in an integrated environment of rules and freedom. Authoritative caregivers justify their actions and let the child take control of their own within fair boundaries. In such a democratic home environment, children are less likely to experience mental health problems [42]. Indeed, previous empirical studies also have provided evidence that authoritative parenting style can help prevent aggression and reduce peer problems among preschool children; in other words, an authoritative parenting style is negatively associated with internalizing, externalizing, and prosocial behavioral problems [40, 43, 46, 47]. In search for reasons that might explain the associations between parenting style and the mental health of young children, according to Baumrind (1967), authoritative parenting style is more sensitive to the needs and skills of children since it is supposed to be based on warmth and support, induction and encouragement, reasoning or guiding, democratic participation, parental responsive attitudes, and proper control. The literature suggests that authoritative parenting style has been shown to be a protective factor for child mental health [31, 58].

While the results in this study concur with the findings that show the negative association between authoritative parenting style and child mental health problems, the analysis also reveals the positive association between the authoritarian parenting style and child mental health problems. These findings regarding the mental health of children with caregivers that use authoritarian parenting styles also is in line with previous studies [15, 31, 41, 58]. According to these studies, an authoritarian parenting style of caregiver, characterized by the lack of warmth and support, undermines the relationship of caregiver-child and causes the child either to exhibit overly submissive behavior that could lead to different types of internalizing problems, or to rebel against the caregiver in various forms of externalizing problems. In such scenarios, an authoritarian parenting style is often shown to be positively associated with child mental health problems. The literature demonstrates that the possible reasons for the effect of authoritarian parenting on child mental health involve the attempts of the caregiver to obtains control overall the child and this can lead to a rejection of the child’s activities, uses punishment and parental enforcement in parenting, and can ultimately leads to anxiety, fear, bewilderment, and dissatisfaction of children which in turn can induce internalizing problems and externalizing problems [38, 41, 57].

In rural China, primary caregivers (especially the grandparents of children), still influenced by Confucianism, tend to stick to traditional parenting strategies. They often place great stress on the parent-child hierarchy, demand respect and obedience from children, and impose more restrictions and disciplines rather than offering affective support [62]. All such interactive activities are more related to an authoritarian parenting style and less related to an authoritative parenting style, which in turn has often been associated with a negative impact on the mental health outcomes of rural children. To mitigate the severity of mental health problems among rural young children, the literature has begun to encourage caregivers to adopt strategies of authoritative parenting style in rural China.

In this period of transition of caregivers in rural China in their selection of parenting styles, more and more parents are beginning to use parenting strategies which combine elements of both authoritative and the authoritarian parenting styles [60–65]. Because of this, our study examines the associations between different subgroups of combined parenting styles and child mental health. When doing so the paper finds that different combinations of parenting styles can have different associations with child mental health problems. Although no previous study of the associations between the combinations of different parenting styles and child mental health has been conducted either in China or outside of China, our findings are consistent with the literature that have shown that the more an authoritative parenting style is used, the lower is the likelihood that a caregiver’s child will have mental health problems [31, 62]. For example, when comparing children of caregivers that use combined parenting styles with low levels of both authoritative and authoritarian parenting styles (Group 4 in our sample), the children of caregivers that use combined parenting styles with high authoritative and low authoritarian (Group 1) are less likely to have externalizing problems and more likely to exhibit prosocial behavior. According to the literature, one possible mechanism might be that the parenting style used by caregivers in Group 1 are dominantly an authoritative strategy, which gives children more warmth which then decreases the possibility of oppositional behavior [48]. In contrast, our study also demonstrates that when caregivers use parenting styles that are more related to authoritarian, their children are more likely to have mental health problems [30, 41, 57]. The underlying reason of these findings might be that parenting styles that involve high levels of warmth, encouragement, support, and lower levels of control, enforcement and punishment are able to improve the atmosphere of the environment in which the child is raised, which in turn affects the overall social development and well-being of the child and ultimately results in lower incidence of mental health problems. If we use an example from the results from our sample, children of caregivers in Group 2 (high authoritarian, low authoritative) are more likely to have high levels of mental health problems. The reason for this may lie in the fact that the dominant parenting strategies of this pattern of combined parenting styles are more likely to be harsh and demanding, which has been shown to increase the likelihood of caregiver-child conflicts that is detrimental to the well-being of children [41, 57]. Even for children of caregivers in Group 3 [high authoritative, high authoritarian), the benefits of the authoritative parenting style could be offsetting the negative effect of the authoritarian parenting style on the outcome of child mental health. These findings might be due to the fact that there is uncertainty of the home environment and this could make children suffer frustrations and struggles (since they are receiving mixed signals).

When investigating the determinants of the adoption of parenting styles, our study identified a number of socio-demographic characteristics, such as age, gender, and the cognitive development of the child, migration status of the parents, and the SES of the family, that are associated with the authoritative and authoritarian parenting styles. The findings in our study are consistent with previous studies that have found that certain demographic characteristics are often correlated with different parenting styles [30, 51, 53, 69]. In terms of child characteristics, parents are reported to show more authoritarian parenting toward boys than girls in our study as well as in the literature [30, 51]. While it is not shown in the paper empirically having such parental expectations on the gender differences, when comparing boys to girls, parents may be more likely to discipline boys (who often are more aggressive) using authoritarian parenting style rather than the authoritative parenting style [56]. Regarding the age of the child, the literature also has shown (as does our study) that younger children are expected to obey rules and norms more often than do older children. Therefore, the literature shows that parents are more likely to adopt the authoritarian parenting style when they are rearing older children [49].

Our findings also show that primary caregivers with higher levels of SES are more likely to adopt the authoritative parenting style. In contrast, primary caregivers with lower levels of SES are more likely to use parenting styles that are relatively more authoritarian. These findings are similar to those in previous studies that were conducted both in urban China and outside of China [50, 52, 53]. The underlying reason of the differences in the adoption of different parenting styles may lie in that more-educated primary caregivers, compared with less-educated peers, are more likely to understand the importance of inductive and rational parenting for child development and value authoritative parenting styles instead of authoritarian parenting styles. In addition, primary caregivers with lower SES often suffer from economic stress that may impede their ability to rear children in ways that are beneficial to children’s well-being. In contrast, primary caregivers with higher levels of SES do not experience such stress and tend to rely on strategies that are more in line with authoritative parenting styles [50].

In light of these findings, it can be suggested that parenting training programs that aim to improve the understanding of primary caregivers regarding choices of different parenting styles can be implemented in rural China. Rural communities should be encouraged to train caregivers to adopt more authoritative parenting styles instead of relying on traditional authoritarian styles through one-on-one parenting guidance, home-visiting services, community-based parenting training, or media-based promotional campaigns. Central and local governments also need to pay attention to ways that can allow them to improve public health services in rural China that will strengthen the capability of the public health system in rural China that will help local doctors and physicians and other health care actors to identify and treat mental health problems of preschool-aged children in an effective and timely fashion.

To the best of our knowledge, this study is the first study conducted in rural China to analyze the associations between parenting styles and mental health of preschool-aged children. Findings of this study offer important insights into the role that parenting style can play in the well-being of young children in rural settings, where children at preschool age have been found to have high prevalence of mental health problems. The study also fills the gap in the literature of the associations between different combinations of parenting styles (i.e., blends of authoritative and the authoritarian parenting styles) and the mental health problems of children that are preschool age. With increasing rates of adoption of combined parenting styles in China, findings of the current study help shed light on a series of new potential research questions that could be explored in this new area.

This study has three limitations. First, since the data used in the current study contained data from only one time period of the study, we can only interpret the results as being “associations” between parenting styles and child mental health, and we are unable to make casual conclusions of the impact of the parenting styles on child mental health due to lack of data on some of the key variables in earlier waves of the survey. Second, the mental health of children and parenting styles of primary caregivers are assessed by care-reported questionnaires, which might lead to inaccuracies in the responses (for a number of reasons) and thus might cause measurement bias. Since the two measurements (i.e. PSDQ and SDQ) have been used widely, many previous studies have also faced this same limitation. There is, thus, a need in this area that observational measures of parenting styles and mental health of children should be included in the data collection procedure. Future research should consider investigating the causality of parenting styles and child mental health as wells as conduct child-based questionnaire on child mental health. Third, since the sample observations in this study were selected from nationally designated poverty counties in northwestern China, we do not consider our results to be statistically representative of the entire country or other rural regions of China. Future studies should continue to expand on the current study by sampling populations from other rural areas in China.

## Conclusion

This study shows that the prevalence of mental health problems among preschool-aged children is high in rural China. The significant and positive association between the authoritative parenting style and the mental health of children is found. In contrast, the authoritarian parenting style is significantly and negatively associated with the mental health of children. Similarly, the more likely it is that authoritative strategies appear in the combined parenting styles, the less likely it is that the child will have mental health problems. In contrast, the more likely it is that authoritarian activities appear in the combined parenting styles, the more likely it will be that the child has the mental health problems. Child age and gender, the status of parental migration, and the SES levels of families are the main determinants of the adoption of different parenting styles. The findings in this paper suggest that it is important to encourage primary caregivers of preschool-aged children to adopt the authoritative parenting styles, or at least use parenting strategies that are related to the authoritative styles, in order to promote child mental health in rural China.

### Electronic supplementary material

Below is the link to the electronic supplementary material.

Supplementary Material 1: Additional file 1 of cutoofs of the three sub-categories for the SDQ subscales

## Data Availability

The data that support the findings of this study are available from the corresponding author upon request (email: tianjingww@163.com).

## Declarations

All caregivers in the study gave written informed consent that allows themselves and their children to participate in the study.

## Abbreviations

SES: Socioeconomic status
PSDQ: Parenting Styles and Dimensions Questionnaire
SDQ: Strengths and Difficulties Questionnaire, WPPSI-IV: Wechsler Preschool and Primary Scale of Intelligence-Fourth Edition
FSIQ: Full-Scale Intelligence Quotient

## References

[1] Colizzi M, Lasalvia A, Ruggeri M (2020). Prevention and early intervention in youth mental health: is it time for a multidisciplinary and trans-diagnostic model for care?. Int J Ment Health Syst.

[2] Polanczyk GV, Salum GA, Sugaya LS, Caye A, Rohde LA (2015). Annual Research Review: a meta-analysis of the worldwide prevalence of mental disorders in children and adolescents. J Child Psychol Psychiatry.

[3] Achenbach TM, Ivanova MY, Rescorla LA, Turner LV, Althoff RR (2016). Internalizing/Externalizing problems: review and recommendations for clinical and Research Applications. J Am Acad Child Adolesc Psychiatry.

[4] Bor W, Dean AJ, Najman J, Hayatbakhsh R (2014). Are child and adolescent mental health problems increasing in the 21st century? A systematic review. Aust N Z J Psychiatry.

[5] Sawyer MG, Arney FM, Baghurst PA, Clark JJ, Graetz BW, Kosky RJ (2001). The Mental Health of Young people in Australia: key findings from the child and adolescent component of the National Survey of Mental Health and Well-Being. Aust N Z J Psychiatry.

[6] Charach A, Mohammadzadeh F, Belanger SA, Ma AE, Lipman EL, McLennan JD (2020). Identification of Preschool Children with Mental Health. J Can Acad Child Adolesc Psychiatry.

[7] Dougherty LR, Leppert KA, Merwin SM, Smith VC, Bufferd SJ, Kushner MR (2015). Advances and directions in Preschool Mental Health Research. Child Dev Perspect.

[8] Vasileva M, Graf RK, Reinelt T, Petermann U, Petermann F (2021). Research review: a meta-analysis of the international prevalence and comorbidity of mental disorders in children between 1 and 7 years. Child Psychol Psychiatry.

[9] Elberling H, Linneberg A, Olsen EM, Goodman R, Skovgaard AM (2010). The prevalence of SDQ-measured mental health problems at age 5–7 years and identification of predictors from birth to preschool age in a Danish birth cohort: the Copenhagen Child Cohort 2000. Eur Child Adolesc Psychiatry.

[10] Klein AM, Otto Y, Fuchs S, Reibiger I, von Klitzing K (2015). A prospective study of behavioral and emotional symptoms in preschoolers. Eur Child Adolesc Psychiatry.

[11] Navarro JB, Fernández M, de la Osa N, Penelo E, Ezpeleta L (2019). Warning signs of preschool victimization using the strengths and difficulties questionnaire: prevalence and individual and family risk factors. PLoS ONE.

[12] Anselmi L, Piccinini CA, Barros FC, Lopes RS (2004). Psychosocial determinants of behaviour problems in Brazilian preschool children. J Child Psychol Psychiat.

[13] Ma Chuan, Jiang L, Chu Lting, Zhang C, cao, Tian Y, Chen Jjin (2022). Mental health problems of preschool children during the COVID-19 home quarantine: a cross-sectional study in Shanghai, China. Front Psychol.

[14] Mellins CA, Xu Q, Nestadt DF, Knox J, Kauchali S, Arpadi S (2018). Screening for Mental Health among Young South African children: the Use of the strengths and difficulties Questionnaire (SDQ). Glob Soc Welf.

[15] Bao P, Jing J, Jin Y, Hu X, Liu B, Hu M (2016). Trajectories and the influencing factors of behavior problems in preschool children: a longitudinal study in Guangzhou, China. BMC Psychiatry.

[16] Guo Y, Zhang YQ, Wu CA, Yin XN, Zhang JY, Wu JB (2021). Bidirectional associations between parenting styles and conduct problems in Chinese preschool children: the Shenzhen Longhua Child Cohort Study. Psychol Health Med.

[17] Li S, Chen K, Liu C, Bi J, He Z, Luo R (2021). Dietary diversity and mental health in preschoolers in rural China. Public Health Nutr.

[18] Luo J, Zou J, Ji M, Yuan T, Sun M, Lin Q (2019). Emotional and behavioral problems among 3- to 5-Year-Olds Left-behind children in Poor Rural areas of Hunan Province: a cross-sectional study. Int J Environ Res Public Health.

[19] Jing J, Yang C, Wang Y, Su X, Du Y. Impact of COVID-19 on Emotional and Behavioral Problems among Preschool Children: A Meta-analysis [Internet]. In Review; 2023 Aug [cited 2024 Jan 16]. Available from: https://www.researchsquare.com/article/rs-3204765/v1. 10.1186/s12887-024-04931-8 PMC11251369 39014321

[20] Ren Y, Yao X, Liu Y, Liu S, Li X, Huang Q (2019). Outdoor air pollution pregnancy exposures are associated with behavioral problems in China’s preschoolers. Environ Sci Pollut Res.

[21] Zheng Y, Zheng X (2015). Current state and recent developments of child psychiatry in China. Child Adolesc Psychiatry Ment Health.

[22] Althoff RR, Verhulst FC, Rettew DC, Hudziak JJ, Van Der Ende J (2010). Adult outcomes of Childhood Dysregulation: a 14-year follow-up study. J Am Acad Child Adolesc Psychiatry.

[23] Arslan İB, Lucassen N, van Lier PAC, de Haan AD, Prinzie P (2021). Early childhood internalizing problems, externalizing problems and their co-occurrence and (mal)adaptive functioning in emerging adulthood: a 16-year follow-up study. Soc Psychiatry Psychiatr Epidemiol.

[24] Narusyte J, Ropponen A, Alexanderson K, Svedberg P (2017). Internalizing and externalizing problems in childhood and adolescence as predictors of work incapacity in young adulthood. Soc Psychiatry Psychiatr Epidemiol.

[25] Bornstein MH, Hahn CS, Haynes OM (2010). Social competence, externalizing, and internalizing behavioral adjustment from early childhood through early adolescence: developmental cascades. Dev Psychopathol.

[26] Conroy MA, Brown WH (2004). Early identification, Prevention, and early intervention with Young children at risk for emotional or behavioral disorders: issues, trends, and a call for action. Behav Disorders.

[27] Mesman J, Koot HM (2001). Early Preschool predictors of Preadolescent Internalizing and Externalizing DSM-IV diagnoses. J AM Acad Child Psy.

[28] Njoroge WFM, Bernhart KP (2011). Assessment of behavioral disorders in Preschool-aged children. Curr Psychiatry Rep.

[29] Aunola K, Nurmi JE (2005). The role of parenting styles in Children’s Problem Behavior: parenting styles in Children’s behavior. Child Dev.

[30] Braza P, Carreras R, Muñoz JM, Braza F, Azurmendi A, Pascual-Sagastizábal E (2015). Negative maternal and paternal parenting styles as predictors of children’s behavioral problems: moderating effects of the child’s sex. J Child Fam Stud.

[31] Querido JG, Warner TD, Eyberg SM (2002). Parenting styles and child behavior in African American families of Preschool Children. J Clin Child Adolesc.

[32] Baumrind D. Effective parenting during the early adolescent transition. Family transitions. Routledge; 2013. pp. 111–63.

[33] Clarke K, Cooper P, Creswell C (2013). The parental overprotection scale: associations with child and parental anxiety. J Affect Disord.

[34] Gar NS, Hudson JL, Rapee RM. Family factors and the development of anxiety disorders. Psychopathology and the family. Elsevier; 2005. pp. 125–45.

[35] Kolip P, Lademann J. Family and health. Familie und Gesundheit In: Hurrelmann K, Razum O, editors[Handbook of health sciences] Handbuch Gesundheitswissenschaften Weinheim: Beltz Juventa. 2012;517–40.

[36] Alizadeh S, Talib MBA, Abdullah R, Mansor M (2011). Relationship between parenting style and children’s behavior problems. Asian Social Sci.

[37] Azman Ö, Mauz E, Reitzle M, Geene R, Hölling H, Rattay P (2021). Associations between parenting style and mental health in children and adolescents aged 11–17 years: results of the KiGGS cohort study (second follow-up). Children.

[38] Baumrind D (1967). Child care practices anteceding three patterns of preschool behavior. Genetic Psychol Monogr.

[39] Robinson CC, Mandleco B, Olsen SF, Hart CH. The parenting styles and dimensions questionnaire (PSDQ). In: Handbook of family measurement techniques. 2001. p. 319–21.

[40] Hanafi H, Thabet AAM (2017). The relationship between parenting styles and Mental Health problems among Preschool Children living in Gaza Strip. EC Psychol Psychiatry.

[41] Thompson A, Hollis C, Richards D (2003). Authoritarian parenting attitudes as a risk for conduct problems. Eur Child Adoles Psy.

[42] Baumrind D (1966). Effects of authoritative parental control on child behavior. Child Dev.

[43] Choe DE, Olson SL, Sameroff AJ (2013). The interplay of externalizing problems and physical and inductive discipline during childhood. Dev Psychol.

[44] Lin X, Liao Y, Li H (2021). Parenting styles and social competence in Chinese preschoolers: a Moderated Mediation Model of Singleton and Self-regulation. Early Educ Dev.

[45] Sahithya BR, Manohari SM, Vijaya R (2019). Parenting styles and its impact on children– a cross cultural review with a focus on India. Mental Health Relig Cult.

[46] Singh S. Parenting style in relation to children’s mental health and self-esteem: a review of literature. Indian J Health Wellbeing. 2017;8(12).

[47] Yamagata S, Takahashi Y, Ozaki K, Fujisawa KK, Nonaka K, Ando J (2013). Bidirectional influences between maternal parenting and children’s peer problems: a longitudinal monozygotic twin difference study. Dev Sci.

[48] Roskam I, Stievenart M, Meunier JC, Noël MP (2014). The development of children’s inhibition: does parenting matter?. J Exp Child Psychol.

[49] Saltalı ND, İmir HM (2018). Parenting styles as a predictor of the Preschool Children’s Social Behaviours. PER.

[50] Cobb-Clark DA, Salamanca N, Zhu A. Parenting Style as an Investment in Human Development. IZA Discussion Paper. 2019; No 9686.

[51] Conrade G, Ho R (2001). Differential parenting styles for fathers and mothers: Differential treatment for sons and daughters. Aust J Psycholo.

[52] Fauziyah R, Salimo H, Murti B (2017). Influence of psycho-socio-economic factors, parenting style, and Sibling Rivalry, on Mental and Emotional Development of Preschool Children in Sidoarjo District. J Maternal Child Health.

[53] September SJ, Rich EG, Roman NV (2016). The role of parenting styles and socio-economic status in parents’ knowledge of child development. Early Child Dev Care.

[54] Someya T, Uehara T, Kadowaki M, Tang SW, Takahashi S (2000). Effects of gender difference and birth order on perceived parenting styles, measured by the EMBU scale, in Japanese two-sibling subjects. Psychiatry Clin Neurosci.

[55] Vyas K, Bano S, Islamia J. Child’s gender and parenting styles. Delhi Psychiatry Journal. 2016;2(19).

[56] McKee L, Erin Roland N, Coffelt AL, Olson R, Forehand C, Massari et al. Harsh Discipline and Child Problem behaviors: the roles of positive parenting and gender. J Fam Viol. 2007;(22):187–96.

[57] Hosokawa R, Katsura T (2018). Role of parenting style in children’s behavioral problems through the transition from Preschool to Elementary School according to gender in Japan. IJERPH.

[58] Hu Q, Feng Q (2022). Parenting style and prosocial behaviour among Chinese preschool children: a moderation model. Early Child Dev Care.

[59] Jia S, Wang L, Shi Y (2014). Relationship between parenting and proactive Versus reactive aggression among Chinese Preschool Children. Arch Psychiatr Nurs.

[60] Chan SM, Bowes J, Wyver S (2009). Parenting style as a context for emotion socialization. Early Educ Dev.

[61] Hu Z, Wu L (2019). The influence of parental styles on Children’s peer Interaction ability in senior class of Kindergarten—By taking research examples of some kindergartens in B City of Anhui Province (in Chinese). J Shaanxi Xueqian Normal Univ.

[62] Li X, Xie J (2017). Parenting styles of Chinese families and children’s social-emotional and cognitive developmental outcomes. Eur Early Child Educ.

[63] Way N, Okazaki S, Zhao J, Kim JJ, Chen X, Yoshikawa H (2013). Social and emotional parenting: Mothering in a changing Chinese society. Asian AM J Psychol.

[64] Wu P, Robinson CC, Yang C, Hart CH, Olsen SF, Porter CL (2002). Similarities and differences in mothers’ parenting of preschoolers in China and the United States. Int J Behav Dev.

[65] Xu Y, Farver JA, Zhang Z, Zeng Q, Yu L, Cai B (2005). Mainland Chinese parenting styles and parent-child interaction. Int J Behav Dev.

[66] Fan J, Chen BB (2020). Parenting styles and coparenting in China: the role of parents and children’s sibling status. Curr Psychol.

[67] Tan TX, Camras LA, Deng H, Zhang M, Lu Z (2012). Family stress, parenting styles, and behavioral adjustment in preschool-age adopted Chinese girls. Early Child Res Q.

[68] Xia X (2020). Parenting style and Chinese children’s school readiness outcomes: the moderating role of socioeconomic status. Child Youth Serv Rev.

[69] Ren L, Pope Edwards C (2015). Pathways of influence: Chinese parents’ expectations, parenting styles, and child social competence. Early Child Dev Care.

[70] Goodman R (2001). Psychometric properties of the strengths and difficulties Questionnaire. J AM Acad Child Psy.

[71] Du Y, Kou J, Coghill D (2008). The validity, reliability and normative scores of the parent, teacher and self report versions of the strengths and difficulties Questionnaire in China. Child Adolesc Psychiatry Ment Health.

[72] Wechsler D. Wechsler preschool and primary scale of intelligence—fourth edition. The Psychological Corporation San Antonio, TX; 2012.

[73] Chen HY, Chen YH, Liao YK, Chen HP, Lynn R (2017). Dysgenic fertility for intelligence and education in Taiwan. Intelligence.

[74] Wang L, Xian Y, Dill SE, Fang Z, Emmers D, Zhang S (2022). Parenting style and the cognitive development of preschool-aged children: evidence from rural China. J Exp Child Psychol.

[75] Attanasio O, Cattan S, Fitzsimons E, Meghir C, Rubio-Codina M (2020). Estimating the production function for human capital: results from a randomized controlled trial in Colombia. Am Econ Rev.

[76] Cunha F, Heckman J (2007). The technology of skill formation. Am Econ Rev.

[77] Cunha F, Heckman JJ (2008). Formulating, identifying and estimating the technology of cognitive and noncognitive skill formation. J Hum Resour.

[78] Zhang H, Qin X (2019). The impact of the parenting style on the formation of adolescent human capital (in Chinese). J Finance Econ.

[79] Winsler A, Madigan AL, Aquilino SA (2005). Correspondence between maternal and paternal parenting styles in early childhood. Early Child Res Q.

