# Supplementary material for: Parenting style and child mental health at preschool age: evidence from rural China
Source: BMC Psychiatry. 2024 Apr 24;24:314. doi: 10.1186/s12888-024-05707-1 (PMC11044564; doi:10.1186/s12888-024-05707-1)
Supplement: Supplementary file 1 — Supplementary Material 1: Additional file 1 of cutoofs of the three sub-categories for the SDQ subscales [file 12888_2024_5707_MOESM1_ESM.docx]

**Appendix**

**Table A1.** Cutoffs of the three sub-categories for the SDQ subscales

|  | Normal range | Borderline range | Abnormal range |
| --- | --- | --- | --- |
| Emotional symptoms | 0-3 | 4 | 5-10 |
| Peer problems | 0-4 | 5 | 6-10 |
| Conduct problems | 0-2 | 3 | 4-10 |
| Hyperactivity | 0-6 | 7 | 8-10 |
| Prosocial behavior | 10-6 | 5 | 4-0 |

*Note*. Adapted from Du et al. (2008).

**Table A2** Pearson linear correlation coefficients

|  | 1 | 2 | 3 | 4 | 5 | 6 | 7 | 8 | 9 | 10 | | 11 | | 12 | |  |
| --- | --- | --- | --- | --- | --- | --- | --- | --- | --- | --- | --- | --- | --- | --- | --- | --- |
| *Child mental health outcome* | | | | | | | | | | | | |  | |  | |
| Total difficulties | 1 |  |  |  |  |  |  |  |  |  | |  | |  | |  |
| *Parenting style* | | | | | | | | | | | | | | | | |
| Authoritative | -0.08** |  |  |  |  |  |  |  |  |  | |  | |  | |  |
| Authoritarian | 0.29*** | 0.02 |  |  |  |  |  |  |  |  | |  | |  | |  |
| *Other characteristics* | | | | | | | | | | | | | | |  | |
| Child age | -0.04 | -0.03 | -0.04 |  |  |  |  |  |  |  | |  | |  | |  |
| Male | 0.06* | -0.04 | 0.06* | -0.02 |  |  |  |  |  |  | |  | |  | |  |
| Has siblings | -0.05 | -0.04 | 0.00 | 0.05 | -0.11*** |  |  |  |  |  | |  | |  | |  |
| Attends preschool | 0.03 | 0.01 | -0.04 | 0.15*** | -0. 05 | -0.03 |  |  |  |  | |  | |  | |  |
| Left-behind child | 0.02 | -0.11*** | 0.16*** | -0.01 | 0.05* | -0.14*** | -0.01 |  |  |  | |  | |  | |  |
| Cognitively delayed | 0.08** | -0.12*** | 0.10*** | -0.08** | 0.06* | 0.06* | -0.14*** | -0.01 |  |  | |  | |  | |  |
| Primary caregiver | -0.04 | 0.12*** | -0.16*** | 0.00 | -0.04 | 0.18*** | -0.02 | -0.69*** | -0.01 |  | |  | |  | |  |
| Caregiver’s age | -0.04 | -0.03 | 0.08** | 0.12*** | 0.02 | -0.04 | 0.03 | 0.16*** | 0.01 | -0.17*** | |  | |  | |  |
| Caregiver’s education | -0.01 | 0.13*** | -0.11*** | -0.10*** | -0.00 | -0.07** | 0.02 | -0.01 | -0.18*** | 0.00 | | -0.36*** | |  | |  |
| Family asset index | -0.05* | 0.17*** | -0.16*** | -0.02 | -0.01 | -0.03 | 0.11*** | -0.12*** | -0.21*** | | 0.05 | -0.04 | | 0.28*** | |  |

**p < .05 **p < .01 *** p<.001*

**Table A3** Associations between primary caregiver’s subscale scores of parenting style and child mental health outcomes (N = 1,459)

|  | Total difficulties | Internalizing problems | Externalizing problems | Prosocial behaviors |
| --- | --- | --- | --- | --- |
| *Subscale scores of authoritative parenting style* | | | | |
| Connection dimension | -1.05*** | -0.44** | -0.61*** | 0.85*** |
|  | (0.23) | (0.14) | (0.14) | (0.08) |
| Regulation dimension | -0.51** | -0.25* | -0.26* | 0.59*** |
|  | (0.18) | (0.11) | (0.11) | (0.07) |
| Autonomy granting dimension | -0.55*** | -0.09 | -0.46*** | 0.59*** |
|  | (0.16) | (0.10) | (0.11) | (0.07) |
| *Subscale scores of authoritarian parenting style* | | | | |
| Physical coercion dimension | 1.58*** | 0.71*** | 0.86*** | -0.21** |
|  | (0.19) | (0.12) | (0.12) | (0.08) |
| Verbal hostility dimension | 1.48*** | 0.63*** | 0.85*** | -0.04 |
|  | (0.17) | (0.11) | (0.11) | (0.08) |
| Non-reasoning/punitive dimension | 1.45*** | 0.92*** | 0.53*** | -0.12 |
|  | (0.20) | (0.12) | (0.13) | (0.08) |
| Controls | Yes | Yes | Yes | Yes |
| Cluster | Yes | Yes | Yes | Yes |
| County fixed effects | Yes | Yes | Yes | Yes |
| Tester fixed effects | Yes | Yes | Yes | Yes |

*Note.* Each cell is a separate regression. Controls include child age, child gender, whether child has siblings, whether child attends preschool, whether child is cognitively delayed, whether child is left-behind (defined as both parents migrate), whether primary caregiver is mother, caregiver’s age, whether caregiver has obtained at least 9 years schooling, and the household asset index.

**p < .05 **p < .01 *** p<.001*

**Table A4** Distribution of groups with different combinations of authoritative and authoritarian parenting styles

| Parenting style | Frequency | Percentage |
| --- | --- | --- |
| Group 1 (High authoritative, low authoritarian) | 363 | 24.88% |
| Group 2 (High authoritarian, low authoritative) | 367 | 25.15% |
| Group 3 (High authoritative, high authoritarian) | 387 | 26.53% |
| Group 4 (Low authoritative, low authoritarian) | 342 | 23.44% |

*Note.* The cutoffs of the classification are the medians of the authoritative and the authoritarian subscale scores.
